# A Comparison Between Intensive and Conventional Therapies: A Systematic Review and Meta-Analysis Regarding the Pre-operative Outcomes After Total Knee Replacement

**DOI:** 10.7759/cureus.75141

**Published:** 2024-12-05

**Authors:** Mohamed Zahed, Alzahraa Faris Alesawy, Ziad Samir Zahed, Ahmed Mohamed, Rahafat Samir, Mahmoud Eleisawy

**Affiliations:** 1 Orthopedics, John Radcliffe Hospital, Oxford University Hospitals NHS Trust, Oxford, GBR; 2 Ophthalmology, Faculty of Medicine, Benha University, Qalubiya, EGY; 3 Ophthalmology, Faculty of Medicine, Benha university, Qalubiya, EGY; 4 Orthopedics, Royal Cornwall Hospital NHS Trust, Truro, Cornwall, GBR; 5 Ophthalmology, Benha University Hospitals, Benha University, Qalubiya, EGY

**Keywords:** meta-analysis, osteoarthritis, pre-operative, systematic review, therapy training, total knee arthroplasty

## Abstract

Joint degeneration characterized by cartilage deterioration and bone wear is the hallmark of osteoarthritis (OA), a condition that worsens over time. Total knee arthroplasty (TKA) is the most common effective treatment for OA. Conventional therapy training (CTT) is the standard intervention; we are testing whether intensive therapy training (ITT) provides different results when used preoperatively. Our study compared intensive and standard preoperative physical therapy in randomized and non-randomized controlled trials, excluding various other study types. Two independent researchers assessed the risk of bias using appropriate tools (RoB 2 for RCTs (Cochrane Methods, London, UK) and ROBINS-I for non-randomized studies (Cochrane Methods, London, UK)). The analysis, conducted using ReviewManager 5.4 (Cochrane Methods, London, UK), presented results as mean differences (MD) with 95% CIs, employing fixed or random-effects models based on heterogeneity assessments.

With a total number of 490 participants, ITT showed significant improvements in the six or 10-minute walk test (MD = 45.07m, P < 0.000001), quadriceps strength (MD = 0.07 Kg, P < 0.0001), range of motion (ROM) flexion (MD = 4.29, P = 0.03), isometric knee flexion (MD =2.32, P=0.04), SF-36 physical component (MD = 1.19, P <,0.0001), stair test (MD = -2.01, P = 0.01), timed up and go test (MD = -1.12, P = 0.02), and Western Ontario and McMaster Universities Osteoarthritis Index (WOMAC) score (MD = -8.43, P = 0.002). Conversely, CTT showed better results in isometric knee extension (MD = 3.45, P = 0.02).

No significant differences were found in ROM extension or visual analog score (VAS) pain scores. Preoperative ITT demonstrates overall superior outcomes compared to CTT for total knee arthroplasty patients. ITT significantly improved various functional and patient-reported outcomes, including walking capacity, quadriceps strength, range of motion, and quality of life measures. However, CTT showed superiority in isometric knee extension. We recommend implementing preoperative ITT protocols for TKA patients while acknowledging the need for further research to optimize exercise specifics, frequency, and duration for optimal results.

## Introduction and background

Joint degeneration characterized by cartilage deterioration and bone wear is the hallmark of osteoarthritis (OA), a condition that worsens over time [1]. This debilitating disorder often manifests as persistent discomfort, potentially leading to impaired mobility and function. The impact of OA extends beyond the physical changes in joint structures, as the chronic pain associated with this condition can significantly compromise an individual's ability to perform daily activities [2].

Epidemiological data indicate that the Americas bear the highest overall OA burden globally, while Asia demonstrates a disproportionately high prevalence of knee-specific OA. To mitigate this substantial health challenge [3], it is needed to implement comprehensive prevention strategies and targeted interventions aimed at addressing modifiable risk factors associated with OA in order to reduce the increase in cases annually [4]. One of the main reasons for the increase in the number of patients suffering from OA is the increase in the number of aged populations with other risk factors, such as obesity and not performing physical activities [5].

Total knee arthroplasty (TKA), typically performed in advanced osteoarthritis cases, offers modest improvements in proprioception with higher safety than other interventions [6,7]. However, some sensory deficits may persist post-surgery [8]. Additionally, patients often experience significant quadriceps weakness on the operated side, with strength potentially reduced by up to 30% compared to the unaffected limb [9]. These strength and balance impairments can lead to various functional issues, including uneven weight distribution between legs, compromised balance, modified movement patterns, and overall reduced physical functionality [9,10]. TKA can be performed using cemented, cementless, or hybrid fixation methods. Previous meta-analyses have indicated no significant differences in clinical outcomes among these fixation techniques, with the availability of these techniques being performed in a conventional way or robotic-assisted [5,11].

Most of the studies use low-intensity training as a therapeutic procedure, although recent research indicates that higher-intensity training protocols may yield superior outcomes for knee rehabilitation [12]. These more vigorous exercise regimens are associated with improved functional strengthening of the knee and enhanced overall knee function [13]. Importantly, these intensive training approaches have been found to maintain safety standards, suggesting they can be effectively implemented without increasing risk to patients [14]. This study's objective is to evaluate and contrast the safety and efficacy of intensive therapy training (ITT) versus conventional therapy training (CTT) programs implemented preoperatively for TKA patients. We seek to provide clear guidance for clinical professionals on optimizing preoperative physical therapy to enhance recovery, improve functional outcomes, and ensure patient safety.

## Review

Methods

We performed our meta-analysis according to the Preferred Reporting Items for Systematic Reviews and Meta-Analyses (PRISMA) checklist and the guidelines that were mentioned in the *Cochrane Handbook for Systematic Reviews and Meta-Analysis* [15,16]. 

Searching Databases and Keywords

We did our search through five databases (PubMed, Cochrane Library, Web of Science, Scopus, and Embase), and it was completed in July 2024. The terms that we used were: (("heavy" OR "progressive" OR "maximal" OR "explosive" OR "resistance" OR "high-intensity" OR "intensified" OR "strengthening" OR "weight lifting" OR "weight bearing" OR "concentric" OR "eccentric" OR "endurance" OR "elastic tube" OR "pulleys") AND ("arthroplasty" OR "replacement" OR "surgery" OR "operation") AND (knee) AND ("training" OR "exercise" OR "rehabilitation") AND ("randomized" OR "randomly" OR "randomised" OR "random")).

Eligibility Criteria and Study Selection

The PICOS (Population, Intervention, Comparison, Outcome, and Study Design) framework was used to guide the development of search terms, strategy, and inclusion criteria for the study [17]. The framework components were defined as follows: the population included individuals who recently underwent knee arthroplasty and performed preoperative exercise; the intervention was pre-operative intensive physical therapy; the comparison was standard pre-operative physical therapy; the outcomes included clinical measures and questionnaire results; and the study designs considered were randomized and non-randomized controlled trials. Only English articles with full texts were included

The study excluded observational research (including cohort studies, case series, and case reports), literature reviews, animal studies, qualitative investigations, and single-arm trials.

Quality Assessment

To maintain data integrity, two independent researchers extracted information from the selected trials. In cases of disagreement, additional authors provided oversight and facilitated consensus. We used two different tools according to the study type: for randomized controlled trials (RCTs), the Cochrane Risk of Bias (RoB) 2 (Cochrane Methods, London, UK) tool was utilized, evaluating five critical domains [18]. Non-randomized studies were assessed using the Risk of Bias in Non-randomized Studies of Interventions (ROBINS-I) tool (Cochrane Methods, London, UK) [19].

Data Extraction

Data extraction was performed independently by two authors using Excel spreadsheets (Microsoft Corporation, Redmond, USA), encompassing three main categories of information. The first category, summary data, included study timing and locations, design, protocol number, total patient count, ITT and CTT details, follow-up duration, and primary outcome. The second category covered baseline patient characteristics such as study groups, age, gender, BMI, height, and knee arthroplasty laterality and location. The third category focused on clinical outcomes, further divided into physical measures (including 6 or 10-minute walk test, quadriceps strength, range of motion for extension and flexion, isometric knee extension and flexion stair test, and Timed Up and Go test) and questionnaires (comprising the Western Ontario and McMaster Universities Osteoarthritis Index (WOMAC) and Visual Analog Scale (VAS) [20].

Data Analysis

The analytical component of the study utilized Review Manager version 5.4 software (Cochrane Methods, London, UK). For continuous outcomes, results were presented as mean differences (MD) with 95% confidence intervals [10]. Statistical significance was established at a p-value threshold of 0.05. Heterogeneity assessment employed χ2 and I-square (I2) tests, with heterogeneity considered present when the χ2 p-value was below 0.1 or the I2 value exceeded 50%. In cases where heterogeneity was detected, a random-effects model was applied; otherwise, a fixed-effects model was used. When heterogeneity persisted despite the use of a random-effects model, a leave-one-out sensitivity analysis was conducted.

Results

Literature Search and Study Selection

**Figure 1:**
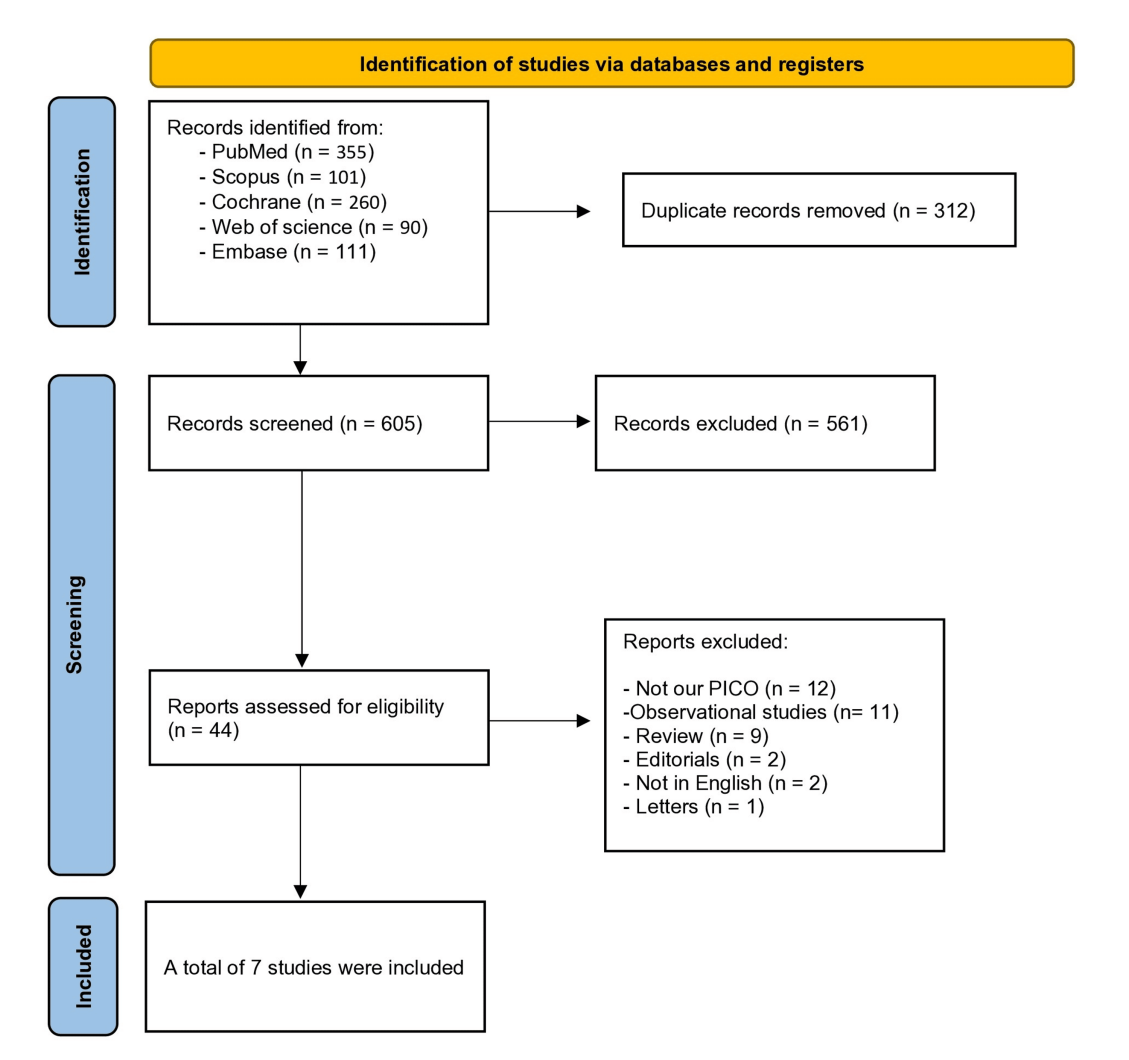
PRISMA flow chart. PRISMA: Preferred Reporting Items for Systematic Reviews and Meta-Analyses

After applying our search strategy, we found a total of 917 articles, and after the removal of the duplicates, a total of 605 studies proceeded for the title and abstract screening. We performed a title and abstract screening that led to the elimination of 561 articles. Finally, after full-text screening, a total of seven studies [20-26] were included. The full, detailed PRISMA flowchart (Preferred Reporting Items for Systematic Reviews and Meta-Analyses) is shown in Figure 1. Details of excluded studies after full-text screening are in the Appendices.

Study Characteristics

Seven studies were included, with a total of 490 participants. The locations of the studies were Japan, China, Switzerland, and Greece. Various rehabilitation programs were used, whether for intensive therapy or the conventional ways. There are some differences regarding the type of exercise, duration, and number of sessions. The follow-up duration ranged from three months to 13 months. Full details about the summary characteristics are shown in Table 1. The majority of the included participants were females and elderly people more than 60 years of age. The BMI of the included population ranged from 29 to 35. The majority of the patients had unilateral knee arthroplasty. Full details about the baseline characteristics are shown in Table 2

**Table 1:** Summary characteristics of the included studies. ADLs: Activities of daily living, NR: Not Reported, PNF: proprioceptive neuromuscular facilitation

Study	Study time and sites	Study design	Protocol number	Total Number of Patients	Intensive therapy details	Conventional therapy details	Follow-up duration (Months)	Primary outcome
Calatayud et al., 2017 [21]	2014	RCT	NR	50	Type; preoperative physical therapy. Duration; 8 weeks Number of sessions: 24 Exercise types: warm-up, strengthening exercise based on five sets of 10 repetitions of each exercise, balancing exercises.	No details	3	1- Western Ontario and McMaster Universities Osteoarthritis Index. 2- Short Form-36 Health Survey 3- Visual Analog Scale
Domínguez‑Navarro et al., 2020 [22]	2017-2019	RCT	NCT02995668	82	Type: Preoperative rehabilitation program Duration: 4 weeks Number of sessions: 12 sessions Exercise types: 1. Strengthening exercises. 2. Colson chair for quadriceps strengthening. 3. Isotonic activation of hamstrings. 4. Colson chair for hamstring strengthening. 5. Lateral Abduction. 6. Adduction. 7. Stretching.	Participants in the control group did not participate in any experimental preoperative intervention.	13	1 The Berg Balance Scale. 2 function in daily living subscale of the Knee Injury and Osteoarthritis Outcome Score.
Gränicher et al., 2020 [23]	Switzerland, 2016-2017	RCT	NCT03160534	20	Type: Preoperative rehabilitation program Duration: 3 to 4 weeks Number of sessions: 5 to 9 Exercise types: Endurance training for 10-45 minutes on a bicycle ergometer, pedal trainer, treadmill, or cross-trainer - PNF techniques for quadriceps and hamstring muscles - Individual interventions: Strengthening exercises targeting muscles of the lower extremities and sensori-motor training consisting of balance exercises on one leg, sessions on how to walk with crouches, and training of physiological movement patterns in activities of daily living (ADLs).	No details.	3	The Stair Climbing Test.
Hashizaki et al., 2023 [24]	Japan, 2018 - 2020	Non-RCT	UMIN 000032568	49	Type; preoperative physical therapy. Duration; 3 weeks. Number of sessions: 90 minutes. Exercise types: warm-up exercises, strengthening exercises. based on five sets /10 repetitions, balancing exercises, single leg stance hand and leg exercises,	Some preoperative exercise instructions (quadriceps strength, landing exercises).	3	The 6-min walking distance
Jiao et al.,2024 [25]	China,2019-2020	RCT	NR	91	Type: preoperative rehabilitation program Duration: preoperative, day of surgery, from 1 to 5th day after surgery. Exercise types: knee bend training, straight leg raising, strengthening training day of surgery; massage of limb, ankle flexion-extension, strength training, straight leg raising, knee bend.	Type; post-operative rehabilitation program (no preoperative rehabilitation program) Time: from 1st day after surgery Duration: 5 days Exercise types: post-operative low-intensity exercises.	12	The American Hospital for Special Surgery Knee Score
Sun et al., 2023 [26]	China,2020–2021	RCT	ChiCTR2000032857	100	Type: preoperative physical therapy Duration: 4 weeks Number of sessions: 20 sessions Exercise types: warm-up, strengthening exercises.	Home preoperative online video training & supervision model for 4 weeks.	12	Knee Society score.
Vasileiadis et al., 2022 [20]	Greece, 2014 -2017	Quasi-experimental trial	NR	98	Preoperative physical therapy and postoperative rehabilitation program	Type; postoperative rehabilitation program only; DURATION: 4 weeks Number of sessions: 5 sessions Exercise types: as the intervention	3	1. Knee Injury and Osteoarthritis Outcome Score 2- Western Ontario and McMaster Universities Osteoarthritis Index.

**Table 2:** Baseline characteristics of the included studies. NR: not reported, ST: strengthening

Study	Study Group	Age, Mean ±SD	Sex (male), No.(%)	BMI, Mean ±SD	Height, Mean ±SD	Knee Arthroplasty		Knee Arthroplasty	
						Unilateral, No. (%)	Bilateral, No. (%)	Left, No.(%)	Right, No.(%)
Catalayud et al., 2017 [21]	High-intensity training (25)	66.8±4.8	NR	32±4.2	1.6±0.1	25 (100%)	0 (0%)	NR	NR
	Control group (25)	66.7±3.1	NR	31±3.8	1.6±0.1	25 (100%)	0 (0%)	NR	NR
Domínguez‑Navarro et al., 2020 [22]	ST Intensive therapy (24)	70.8±5.4	10 (41.7%)	29	1.6±0.04	NR	NR	NR	NR
	ST and balance Intensive therapy (20)	70.4±6.4	7 (35%)	29.7	1.6±0.05	NR	NR	NR	NR
	Conventional therapy (21)	70.2±5.6	7 (33.3%)	29.7	1.6 ±0.06	NR	NR	NR	NR
Gränicher et al., 2020 [23]	Intensive therapy (10)	66.6 ±7.52	7 (70%)	29.7	1.7 ±0.11	10 (100%)	0 (0%)	NR	NR
	Conventional therapy (10)	68.1 ±7.68	5 (50%)	28.5	1.6±0.06	10 (100%)	0 (0%)	NR	NR
Hashizaki et al., 2023 [24]	Post-operative rehabilitation group (14)	73.7±4.7	9 (64.2%)	25.1±3.2	1.55±10.3	11 (78.5%)	3 (21.4%)	NR	NR
	Control group (19)	77.7±6.9	16.(84,2%)	22.8±6.3	1.53±7.7	4 (78.9%)	4 (21%)	NR	NR
Jiao et al., 2023 [25]	High-intensity progressive rehabilitation (39)	75±4.43	7.(17.9%)	30±3.3	NR	39 (100%)	0(0%)	NR	NR
	Routine rehabilitation (39)	76±4.43	4.(10.3%)	30±3.3	NR	39 (100%)	0(0%)	NR	NR
Sun et al., 2023 [26]	High-intensity strength training (32)	66.4±8.3	9.(28.1%)	22.6±3.3	NR	32 (100%)	0(0%)	17 (53.1%)	15 (46.8%)
	Control group (32)	68.5±7.9	12.(34.2%)	23.6±2.5	NR	32 (100%)	0(0%)	16 (45.7%)	19 (54.2%)
Vsileidis et al., 2022 [20]	High-Intensity Preoperative Physiotherapy (44)	68.7±5.2	20.(45.4%)	31±4.1	NR	44 (100%)	0(0%)	NR	NR
	Control group (44)	68.9±5.4	18.(40.9%)	30±3.9	NR	44 (100%)	0(0%)	NR	NR

Quality of the Included Studies

**Figure 2:**
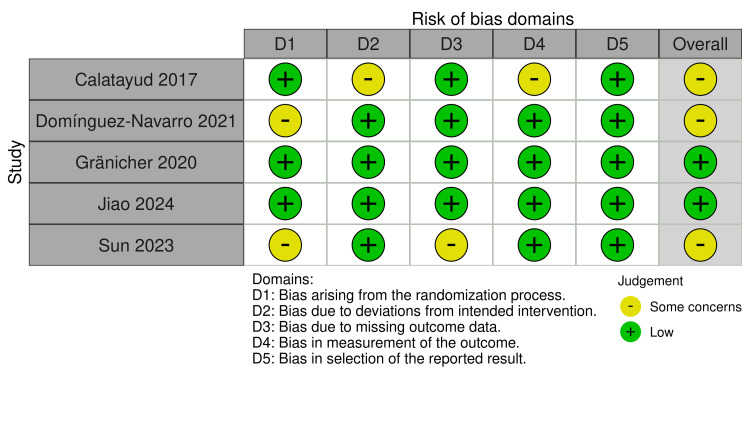
Risk of bias graph summary for RCTs made by the RoB 2 tool. Catalayud et al., 2017 [21], Domínguez‑Navarro et al., 2020 [22], Gränicher et al., 2020 [23], Jiao et al., 2024 [25], and Sun et al., 2023 [26].

**Figure 3:**
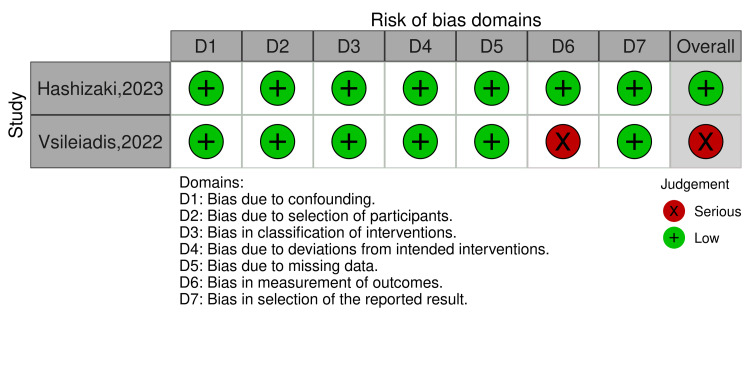
Risk of bias graph summary for non-RCTs made by the ROBINS-I tool Vasileiadis et al., 2022 [20], Hashizaki et al., 2023 [24]

The risk of bias assessment using the RoB 2 tool revealed varying levels of potential bias across the included studies. No studies demonstrated a high risk of bias. However, three studies [21,22,26] were associated with some concerns due to randomization domain or others as deviation from the intended intervention or missing outcome domain. The remaining two RCTs [23,25] were associated with a low risk of bias. A comprehensive visual representation of these assessments is provided in Figure 2. On the other hand, regarding the ROBINS-I, one study showed a low risk of bias, and the other showed a serious risk due to bias in the measurement of the outcomes domain (Figure 3).

Outcomes

Physical Measurements

**Figure 4:**
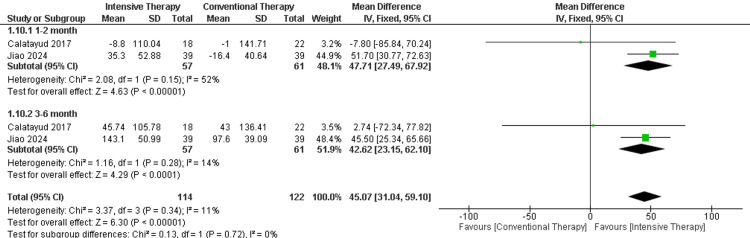
Forest plot of six or 10-minute walk test. Catalayud et al., 2017 [21], Jiao et al., 2024 [25]

Six- or 10-minute walk test (meters): In the follow-up of one to two months, two studies were included, and the result showed a significant increase in the distance that patients were able to walk in the ITT when compared to CTT with results of (MD = 47.71, 95% CI)(27.49 to 67.92], p-value < 0.00001). Moreover, in the follow-up period of three to six months, there were significant differences between the two comparators (MD = 42.62, 95% CI (23.15 to 62.10), p-value < 0.0001). The overall results demonstrated a significant difference favoring ITT, and pooled results were (MD = 45.07, 95% CI (31.04 to 59.1), P-value < 0.000001). The overall results of the study were homogenous as p-value = 0.34 and I2 =11% (Figure 4).

**Figure 5:**
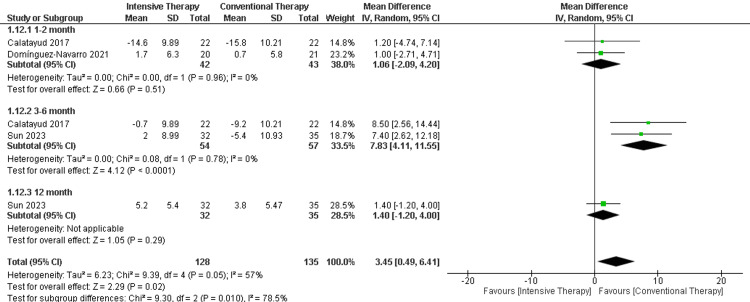
Forest plot of isometric knee extension. Catalayud et al., 2017 [21], Domínguez‑Navarro et al., 2020 [22], Sun et al., 2023 [26].

Isometric knee extension (Kg): In the follow-up period ranging from one to two months, there was no significant difference in the isometric knee extension (MD = 1.06, 95% CI (-2.09 to 4.20), P-value = 0.51). Additionally, the follow-up period ranging from three to six months showed a significance favoring CTT over ITT (MD = 7.83, 95% CI (4.11 to 11.55), p-value < 0.0001). Regarding the long-term follow-up (12 months), only one study was included, with no significant difference between the comparators. The overall results favored the CTT over ITT (MD = 3.45, 95% CI (0.49 to 6.41), p-value = 0.02) (Figure 5).

**Figure 6:**
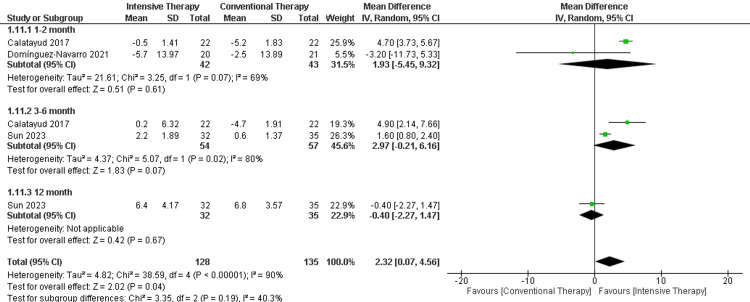
Forest plot of isometric knee flexion. Catalayud et al., 2017 [21], Domínguez‑Navarro et al., 2020 [22], Sun et al., 2023 [26].

Isometric knee flexion (Kg): In the follow-up period ranging from one to two months, there was no significant difference in the isometric knee flexion. Additionally, the follow-up period ranging from three to six months showed no significant difference between CTT and ITT (MD = 2.97, 95% CI (-0.21 to 6.16), p-value =0.07). Regarding the long-term follow-up (12 months), only one study was included with no significant difference between the comparators. The overall results favored the ITT significantly over CTT (MD = 2.32, 95% CI (0.07 to 4.56), p-value = 0.04). The results were heterogeneous (p-value< 0.00001) and I2=90% ( Figure 6).

**Figure 7:**
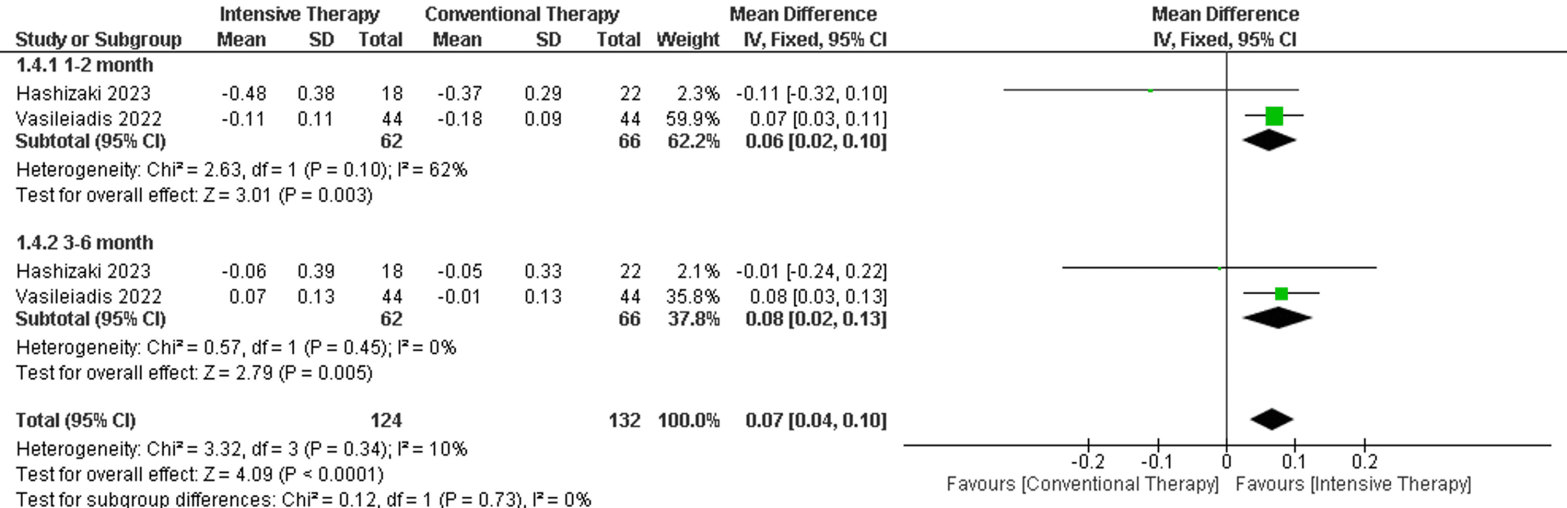
Forest plot of quadriceps strength. Vasileiadis et al., 2022 [20], Hashizaki et al., 2023 [24],

Quadriceps strength (Kg): In the follow-up period ranging from one to two months, there was a significant difference between ITT and CTT regarding the strength (MD = 0.06, 95% CI (0.02 to 0.10), P-value = 0.0003). The follow-up period ranging from three to six months showed another significant difference favoring ITT (MD = 0.08, 95% CI (0.02 to 0.13), p-value =0.005). The overall results favored the ITT over CTT (MD = 0.07, 95% CI (0.07 to 0.10), p-value <0.0001). The results were heterogeneous (p-value< 0.00001) and I2=90% (Figure 7).

**Figure 8:**
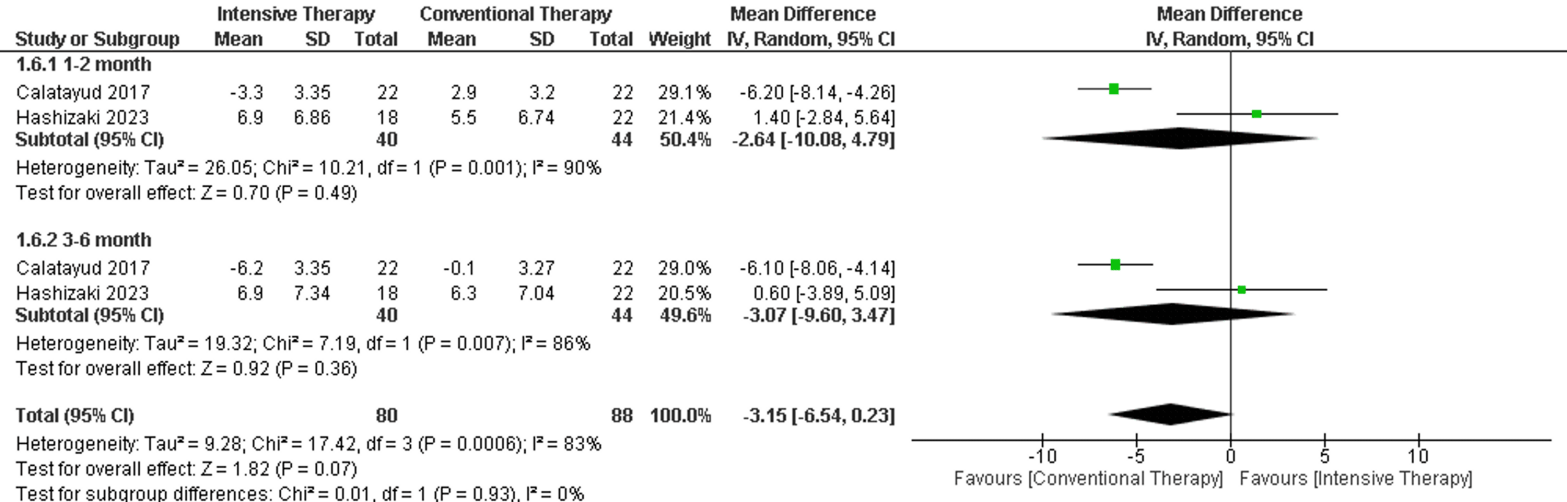
Forest plot of ROM extension. ROM: range of motion. Catalayud et al., 2017 [21], Hashizaki et al., 2023 [24].

ROM extension: In the follow-up period ranging from one to two months, there was no significant difference between ITT and CTT regarding the extension. The follow-up period ranging from three to six months showed another insignificant difference between the two comparator groups. The results were heterogeneous (p-value= 0.00006) and I2=83% (Figure 8).

**Figure 9:**
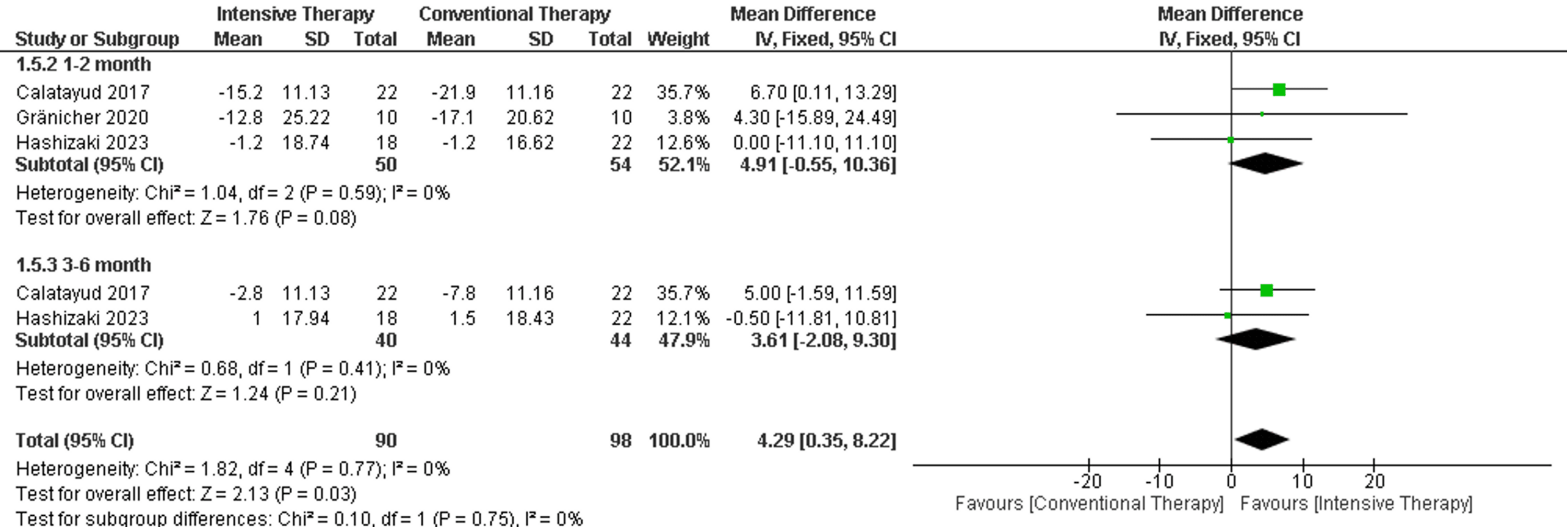
Forest plot of ROM flexion. ROM: range of motion. Catalayud et al., 2017 [21], Gränicher et al., 2020 [23], Hashizaki et al., 2023 [24],

ROM flexion: In the follow-up period ranging from one to two months, there was no significant difference between ITT and CTT regarding the flexion. The follow-up period ranging from three to six months showed another insignificant difference between the two comparator groups. Finally, the overall result showed that ITT was significantly improved in comparison to CTT (MD = 4.29, 95% CI (0.35 to 8.22), p-value = 0.03). The results were homogenous (p-value= 0.77) and I2=0% (Figure 9).

**Figure 10:**
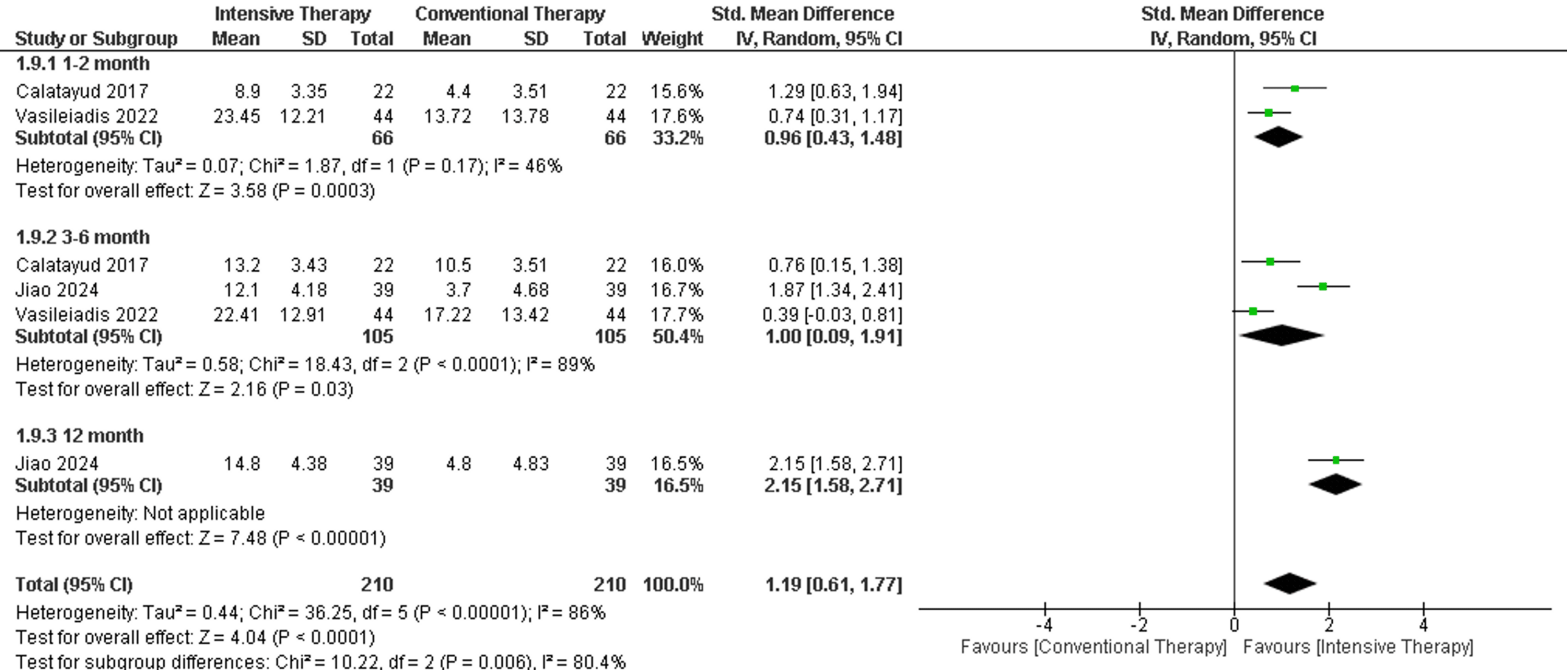
Forest plot of SF-36 physical component SF: Short Form Health Vasileiadis et al., 2022 [20], Catalayud et al., 2017 [21], Jiao et al., 2024 [25]

SF-36 physical component: In the follow-up period ranging from one to two months, two studies were included showing a significant difference in the SF-36 (MD = 0.96, 95% CI (0.43 to 1.48), p-value = 0.0003). Additionally, the follow-up period ranging from three to six months showed a significant difference between CTT and ITT (MD = 1.00, 95% CI (0.09 to 1.91), p-value =0.03). Regarding the long-term follow-up (12 months), only one study was included with a significant difference between the comparators. The overall results favored the ITT over CTT (MD = 2.32, 95% CI (0.07 to 4.56), p-value = 0.04). The results were heterogeneous (P-value < 0.00001) and I2=90% (Figure 10).

**Figure 11:**
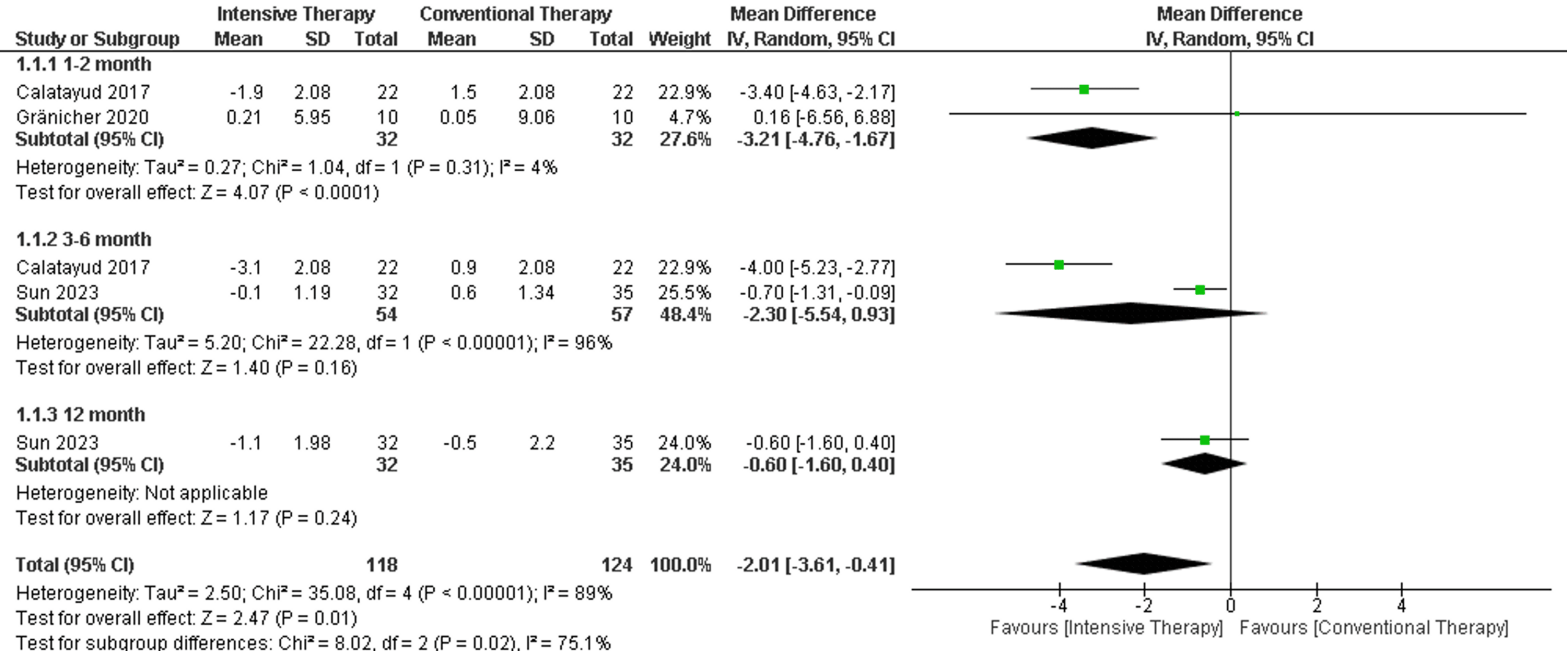
Forest plot of the stair test (seconds). Catalayud et al., 2017 [21], Gränicher et al., 2020 [23], Sun et al., 2023 [26].

Stair test (seconds): In the follow-up period ranging from one to two months, two studies were included showing a significant difference in the stair test (MD = -3.21, 95% CI (-4.76 to -1.67), p-value < 0.0001). Additionally, the follow-up period ranging from three to six months showed a non-significant difference between CTT and ITT (MD = -2.30, 95% CI (-5.54 to 0.93), p-value =0.16). Regarding the long-term follow-up (12 months), only one study was included with a non-significant difference between the comparators. The overall results favored the ITT over CTT (MD = -2.01, 95% CI (-3.61 to -0.41), p-value = 0.01). The results were heterogeneous (p-value< 0.00001) and I2=89% (Figure 11).

**Figure 12:**
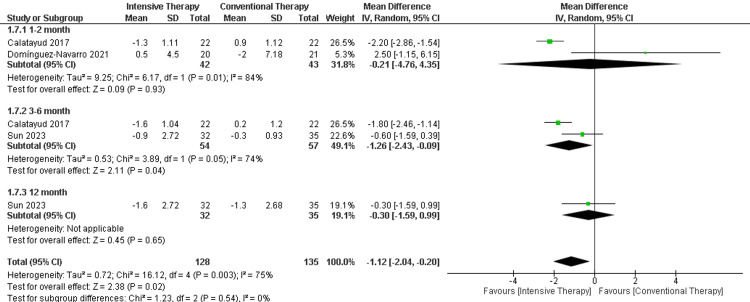
Forest plot of timed up and go. Catalayud et al., 2017 [21], Domínguez‑Navarro et al., 2020 [22], Sun et al., 2023 [26].

Timed up and go: In the follow-up period ranging from one to two months, two studies were included showing a non-significant difference in the time up and go (MD = -0.21, 95% CI (-4.76 to -4.35), p-value =0.93). Additionally, the follow-up period ranging from three to six months showed a significant reduction in time favoring ITT (MD = -1.26, 95% CI (-2.43 to -0.09), p-value =0.04). Regarding the long-term follow-up (12 months), only one study was included with a non-significant difference between the comparators. The overall results favored the ITT over CTT (MD = -1.12, 95% CI (-2.04 to -0.20), p-value = 0.02). The results were heterogeneous (p-value= 0.003) and I2=75% (Figure 12).

Questionnaires

**Figure 13:**
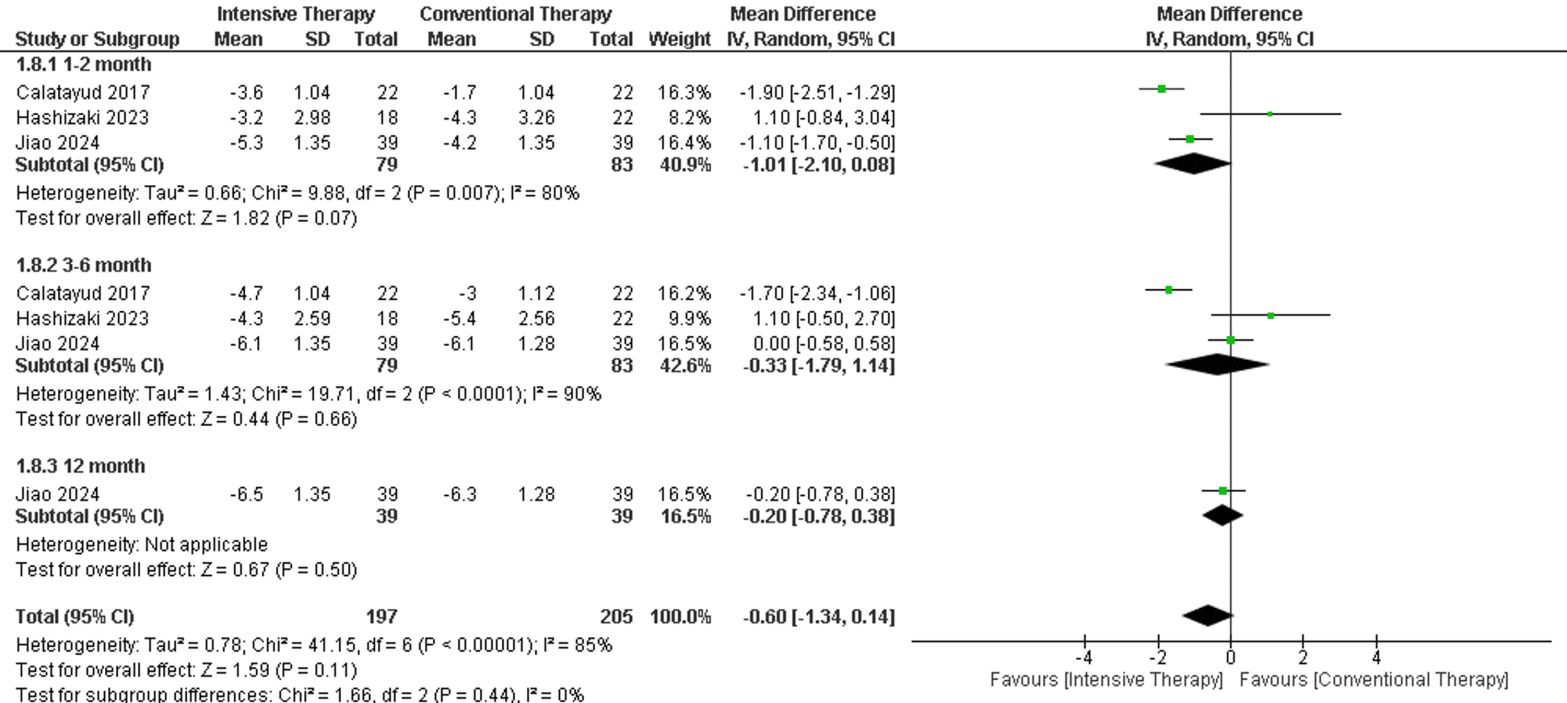
Forest plot of the Visual Analog Score (VAS) score. Catalayud et al., 2017 [21], Hashizaki et al., 2023 [24], Jiao et al., 2024 [25]

VAS score: No results were found significant according to the included studies at the VAS score results. In the follow-up period, which ranged from one month to two months, three studies were included, and the result showed a non-significant decrease in pain in the ITT on VAS when compared to CTT. Additionally, in the follow-up ranging from three to six months, there were no significant differences between the two comparators with three included papers. The long-term follow-up of 12 months was only mentioned in one study without any significant difference. The results of the outcome were heterogeneous as p-value <0.0001 and I2 = 85% (Figure 13).

**Figure 14:**
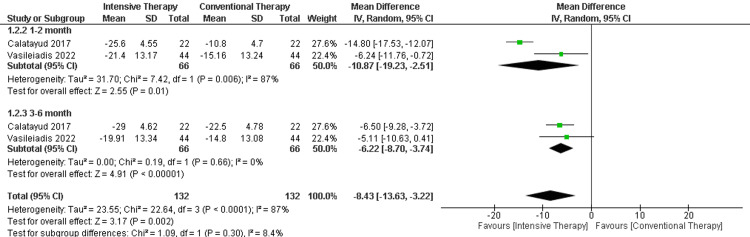
Forest plot of the WOMAC score. WOMAC: Western Ontario and McMaster Universities Osteoarthritis Index Vasileiadis et al., 2022 [20], Catalayud et al., 2017 [21].

WOMAC score: Results showed a significant difference according to the included studies on WOMAC score. In the follow-up period that ranged from one to two months, two studies were included, and the result showed a significant difference in the ITT on WOMAC score when compared to CTT (MD = -10.87, 95% CI (-19.23 to -2.51), p-value = 0.01). Additionally, in the follow-up ranging from three to six months, there was a significant difference between the two comparators with two included papers (MD = -6.22, 95% CI (-8.70 to -3.74), p-value <0.00001). The overall results showed a significant difference in favoring ITT over CTT (MD = -8.43, 95% CI (-13.63 to -3.22), p-value = 0.28). The results of the outcome were heterogeneous as P-value < 0.0001 and I2 = 87% (Figure 14).

Discussion

This systematic review and meta-analysis analyzed seven studies comparing ITT to CTT for knee arthroplasty patients. The study populations were predominantly female and elderly, with no weight specification. The results demonstrated that ITT generally outperformed CTT across several outcomes. Significant improvements were observed in walking tests, quadriceps strength, range of motion flexion, physical component scores, stair tests, timed up and go tests, and WOMAC scores. These benefits were noted across various follow-up periods, ranging from short-term to medium-term and long-term post-intervention. However, the results showed CTT showed better outcomes in isometric knee extension, particularly in the medium-term follow-up period

The selection of appropriate training intensity and duration is critical for optimizing muscle strength gains. While many previous studies focused on low to moderate-intensity regimens [27], recent research has highlighted the potential benefits of higher-intensity approaches [28,29]. Notably, a randomized controlled trial demonstrated that preoperative high-intensity strength training can yield significant improvements. This intensive training was found to reduce pain levels, significantly increase lower limb muscle strength, and enhance performance in functional tasks, suggesting that higher-intensity protocols may offer superior outcomes for patients preparing for knee surgery [30]. Rooks et al. found that preoperative progressive resistance training improved postoperative functional performance and muscle strength but did not significantly affect patient-reported outcomes [31]. This suggests a potential disconnect between objective physical improvements and patients' subjective experiences.

A comprehensive approach to TKA rehabilitation should integrate both preoperative and postoperative physiotherapy programs [21]. Preoperative training for patients with end-stage osteoarthritis has been shown to enhance initial outcomes and improve patient satisfaction following TKA [32]. Conversely, postoperative exercise regimens primarily focus on pain management, edema reduction, and improvement of knee range of motion. This dual-phase approach aims to optimize overall patient outcomes by addressing both preoperative conditioning and postoperative recovery needs [21,33].

Our study has several strengths. By including only RCTs and non-randomized controlled trials, we ensured the inclusion of high-quality evidence, excluding observational studies to minimize potential biases. Furthermore, our study is the first meta-analysis to focus specifically on the preoperative differentiation between ITT and CTT, addressing a critical gap in the literature. The key guidance from our findings highlights that preoperative physical therapy tailored to intensity levels has significant implications for optimizing TKA outcomes. Specifically, the results suggest that ITT protocols may be more effective in improving functional recovery and postoperative strength, while CTT approaches might still hold value in specific patient populations or settings where ITT is less feasible.

Our study also had some limitations, as long-term follow-up (12 months) was limited, with only two studies reporting outcomes at this time point [25,26]. Moreover, various training programs were introduced with different intervention settings which could add possible heterogeneity and make the results affected. The included studies exhibited considerable variation in exercise training protocols which were observed in the duration of training periods, frequency of sessions, and intensity of exercises. This diversity in intervention approaches may have contributed to heterogeneity among the study populations, potentially influencing the comparability and generalizability of results across studies. It's important to note that although ITT generally appears more effective, individual responses to therapy may vary as there is a high heterogenicity among the included studies. Additionally, taking into consideration that some outcomes had no significant difference in the specified follow-up durations and gave overall significance according to the analysis. Previous meta-analyses have suggested that robotic-assisted TKA may offer certain advantages over conventional surgical techniques, particularly in terms of knee alignment and functional outcomes which may add another factor to be considered for investigation and increase the requirement of having a homogenous population [34].

We recommend more long-term follow-up studies beyond 12 months, which are crucial to assess the sustained benefits of intensive therapy compared to conventional approaches. Given the observed heterogeneity in outcomes, future studies should aim to identify patient subgroups that benefit most from intensive therapy, enabling more personalized rehabilitation approaches. Additionally, economic analyses should evaluate the cost-effectiveness of intensive therapy, considering both short-term resource utilization and long-term functional outcomes. Clinical guidelines for post-operative care in TKA should be reviewed and potentially updated to reflect these benefits. Finally, patient education materials should be developed to inform individuals about the potential advantages of intensive therapy and encourage adherence to more rigorous rehabilitation protocols. Special training programs should be made to fit every participant, considering their health and condition.

## Conclusions

We conclude that ITT generally demonstrates overall superior outcomes compared to CTT for patients undergoing knee arthroplasty when done preoperatively. ITT showed significant improvements across a range of functional and patient-reported outcomes, including walking capacity, quadriceps strength, range of motion, and quality of life measures. On the other hand, the isometric knee extension showed superiority for the CTT group. We recommend that healthcare providers should consider implementing preoperative intensive therapy protocols for TKA patients due to their superior outcomes across multiple recovery domains. However, further research is needed to optimize these protocols by focusing on specific exercises, frequency, and duration for the best results.

## Appendices

**Table 3:** List of excluded studies after full-text screening ACL: Anterior Cruciate Ligament; PICO: Population, Intervention, Comparison, Outcome

Study Title	Authors	Year	Study design	Reason for Exclusion
Follow-up of individualised physical activity on prescription and individualised advice in patients with hip or knee osteoarthritis: A randomised controlled trial	Bendrik RK, Bröms LV, Emtner K	2024	RCT	Not our PICO
The effects of high-intensity versus low-intensity resistance training on leg extensor power and recovery of knee function after ACL-reconstruction	Bieler TS, Andersen NA, Kiel P, Løfholm P, Aagaard P, Magnusson SP, Krogsgaard MR, Beyer N	2014	RCT	Not our PICO
The Effect of Progressive Resistance Exercise on Knee Muscle Strength and Function in Participants with Persistent Hamstring Deficit Following ACL Reconstruction: A Randomized Controlled Trial	Bregenhof BA, Nissen P, Creaby MW, Thorlund JB, Jensen C, Torfing T, Holsgaard-Larsen A	2023	RCT	Not our PICO
The effect of targeted exercise on knee-muscle function in patients with persistent hamstring deficiency following ACL reconstruction - study protocol for a randomized controlled trial	Bregenhof BJ, Aagaard P, Nissen U, Creaby MW, Thorlund JB, Jensen C, Torfing T, Holsgaard-Larsen A	2018	RCT	Not our PICO
Low- Versus High-Intensity Plyometric Exercise During Rehabilitation After Anterior Cruciate Ligament Reconstruction	Chmielewski TLG, Tillman SZ, Moser SM, Lentz TA, Indelicato PA, Trumble TN, Shuster JJ, Cicuttini FM, Leeuwenburgh C	2016	RCT	Not our PICO
Feasibility and Preliminary Outcomes of a Physical Therapist-Administered Physical Activity Intervention After Total Knee Replacement	Christiansen MBT, Master LM, Voinier H, Schmitt D, Ziegler LA, LaValley ML, White DK	2020	RCT	Not our PICO
Blood Flow Restriction Training Applied With High-Intensity Exercise Does Not Improve Quadriceps Muscle Function After Anterior Cruciate Ligament Reconstruction: A Randomized Controlled Trial	Curran MTB, Mendias A, Wojtys CL, Kujawa EM, Palmieri-Smith MV	2020	RCT	Not our PICO
High-intensity versus low-intensity resistance training in patients with knee osteoarthritis: A randomized controlled trial	de Zwart AHD, Roorda J, van der Esch LD, Lips M, van Schoor P, Heijboer NM, Turkstra AC, Gerritsen F, Häkkinen M, Bennell A, Steultjens MP, Lems WF, van der Leeden M	2022	RCT	Not our PICO
Relaxation exercise therapy improves pain, muscle strength, and kinesiophobia following total knee arthroplasty in the short term: a randomized controlled trial	Eymir MU, Karatosun B	2022	RCT	Not our PICO
Effects of high-velocity resistance training on muscle function, muscle properties, and physical performance in individuals with hip osteoarthritis: a randomized controlled trial	Fukumoto YT, Ikezoe H, Tsukagoshi T, Akiyama R, So H, Kuroda K, Ichihashi N	2014	RCT	Not our PICO
Postoperative effects of progressive resistance training prior to total hip arthroplasty e one-year outcome of a randomized controlled trial	Holsgaard-Larsen AH, Zerahn A, Mejdahl B, Overgaard S	2018	RCT	Not our PICO
Predictors of Moderate-Vigorous Physical Activity Following a Physical Therapist Led Physical Activity Intervention for Adults with Total Knee Replacement	Jamieson SP	2022	RCT	Not our PICO
Rehabilitation after anterior cruciate ligament reconstruction: a systematic review	Kruse LM, Gray B, Wright RW	2012	Review	Review
Rehabilitation protocols following total knee arthroplasty: a review of study designs and outcome measures	Dávila Castrodad IM, Recai TM, Abraham MM, Etcheson JI, Mohamed NS, Edalatpour A, Delanois RE	2019	Review	Review
Anterior cruciate ligament strain and tensile forces for weight-bearing and non-weight-bearing exercises: a guide to exercise selection	Escamilla RF, Macleod TD, Wilk KE, Paulos L, Andrews JR	2012	Review	Review
Knee Osteoarthritis: Alternative Range of Motion Treatment	Benner RW, Shelbourne KD, Bauman SN, Norris A, Gray T	2019	Review	Review
Rehabilitation and return to play after anatomic anterior cruciate ligament reconstruction	Yabroudi MA, Irrgang JJ	2013	Review	Review
Physical exercise after knee arthroplasty: a systematic review of controlled trials	Pozzi F, Snyder-Mackler L, Zeni J	2013	Review	Review
A systematic review of anterior cruciate ligament primary repair rehabilitation	Hourston GJM, Kankam HKN, McDonnell SM	2022	Review	Review
The preventive and therapeutic role of physical activity in knee osteoarthritis	Restuccia R, Ruggieri D, Magaudda L, Talotta R	2022	Review	Review
Editorial Commentary: Gait Symmetry After Anterior Cruciate Ligament Reconstruction Is Improved Using Functional Rehabilitation Braces That Resist Knee Motion	Rocchi JE	2022	Comment	Editorial
Coper Classification Early After Anterior Cruciate Ligament Rupture Changes With Progressive Neuromuscular and Strength Training and Is Associated With 2-Year Success: The Delaware-Oslo ACL Cohort Study	Thoma LM, Grindem H, Logerstedt D, Axe M, Engebretsen L, Risberg MA, Snyder-Mackler L	2019	Cohort	Observational studies
Feasibility of progressive strength training shortly after hip fracture surgery	Overgaard J, Kristensen MT	2013	Cohort	Observational studies
The safety and effectiveness of bone marrow concentrate injection for knee and hip osteoarthritis: a Canadian cohort	Burnham R, Smith A, Hart D	2021	Cohort	Observational studies
Early high-intensity rehabilitation following total knee arthroplasty improves outcomes	Bade MJ, Stevens-Lapsley JE	2011	Cohort	Observational studies
Occupation and risk of knee osteoarthritis and knee replacement: A longitudinal, multiple-cohort study	Perry TA, Wang X, Gates L, Parsons CM, Sanchez-Santos MT, Garriga C, Cooper C, Nevitt MC, Hunter DJ, Arden NK	2020	Cohort	Observational studies
Does Extended Preoperative Rehabilitation Influence Outcomes 2 Years After ACL Reconstruction? A Comparative Effectiveness Study Between the MOON and Delaware-Oslo ACL Cohorts	Failla MJ, Logerstedt DS, Grindem H, Axe MJ, Risberg MA, Engebretsen L, Huston LJ, Spindler KP, Snyder-Mackler L	2016	Cohort	Observational studies
Treatment after anterior cruciate ligament injury: Panther Symposium ACL Treatment Consensus Group	Diermeier T, Rothrauff BB, Engebretsen L, Lynch AD, Ayeni OR, Paterno MV, Xerogeanes JW, Fu FH, Karlsson J, Musahl V, Svantesson E, Hamrin Senorski E, Rauer T, Meredith SJ; Panther Symposium ACL Treatment Consensus Group	2020	Cohort	Observational studies
Factors affecting knee abduction during weight-bearing activities in individuals with anterior cruciate ligament reconstruction	Cronström A, Ageberg E, Franettovich Smith MM, Blackmore T, Nae J, Creaby MW	2019	Cross-sectional study	Observational studies
Concentric and eccentric torque of the hip musculature in individuals with and without patellofemoral pain	Boling MC, Padua DA, Creighton RA	2009	Case control	Observational studies
Quadriceps and hamstrings muscle dysfunction after total knee arthroplasty	Stevens-Lapsley JE, Balter JE, Kohrt WM, Eckhoff DG	2010	Cohort	Observational studies
Combination of eccentric exercise and neuromuscular electrical stimulation to improve biomechanical limb symmetry after anterior cruciate ligament reconstruction	Lepley LK, Wojtys EM, Palmieri-Smith RM	2015	Case control	Observational studies
